# ID 359 - Aplicação do *Flashcard* Digital como Ferramenta na Formação e Educação Continuada em Terminologia da Avaliação em Tecnologia em Saúde (ATS)

**DOI:** 10.5327/2237-9622.2025.v34s1.359

**Published:** 2025-11-25

**Authors:** Silvio Tacara, Lucimara Santos, Natalia Facaro Lombardi Asinelli, Otilia Beatriz Maciel da Silva, Adeli Regina Prizybicien, Maria Marcia Ferreira, Irene Tomoko Nakano

## Abstract

**Introdução:**

O, ou cartão de memória, é um recurso educacional que expõe uma palavra ou imagem que pode ser utilizado como auxílio no ensino ou na aprendizagem (Oxford English Dictionary, 2022). O principal objetivo de sua utilização é o autoaprendizado e a memorização do seu conteúdo. O modelo básico de estudo com cartões foi idealizado inicialmente na década de 1970. O estudo com tem como base a técnica de repetição espaçada, que se fundamenta na ideia de que "a ativação contínua reforça o circuito neural e torna mais fácil a posterior evocação da informação armazenada"(Mourão, Faria, 2015). Esse processo se faz necessário uma vez que a memória humana é suscetível ao esquecimento ao longo do tempo. De acordo com Nielsen (2018), a probabilidade de relembrar corretamente um item decai exponencialmente com o passar do tempo. A utilização do digital tem como finalidade auxiliar e contribuir na formação e na educação continuada em terminologia da ATS para identificar os termos próprios da área técnica; fornecer referências para a compreensão de termos e conceitos; proporcionar a exatidão conceitual e definir a atuação de cada termo em seus diferentes contextos institucionais; eliminar ambiguidades para facilitar a comunicação interna. O projeto está em fase de finalização e consiste na utilização do Anki Flaschcards, que é um aplicativo com versões para os sistema Android (Ankidroid), IOS (Anki mobile), Windows e Linux. O programa trabalha com revisões espaçadas através dos . Por meio do sistema SRS (), os têm suas revisões agendadas com base em um algoritmo programado para retornar os cartões em determinado prazo, diferenciando os intervalos de revisão para cada cartão de acordo com o nível de dificuldade do usuário em responder à pergunta ou questão elaborada. O formato consistirá em uma apresentação Ignite Talk sobre o projeto.

**Método:**

Apresentação presencial do projeto no formato Ignite Talk. Demonstração do aplicativo Anki Flaschcards e a aplicação na formação e educação continuada em ATS. Sinal de wi-fi, software Anki Flaschcards, um aparelho de multimídia, um notebook ou PC, monitor para apresentação de eslaides, teclado, cabo HDI, adaptador/VGA/HDI, passador de eslaides.
